# Supplementary material for: A Critical View of the Application of the APEX Software (Aqueous Photochemistry of Environmentally-Occurring Xenobiotics) to Predict Photoreaction Kinetics in Surface Freshwaters
Source: Molecules. 2019 Dec 18;25(1):9. doi: 10.3390/molecules25010009 (PMC7017383; doi:10.3390/molecules25010009)
Supplement: Supplementary file 1 [file molecules-25-00009-s001.zip › 3.2.4_gcc-4.4.0.zip!/3.2.4_gcc-4.4.0/3.2.4_gcc-4.4.0/doc/octave/html/interpreter/Optimization.html]

Optimization - Untitled


Next: Statistics,
Previous: Differential Equations,
Up: Top


---

## 24 Optimization

Octave comes with support for solving various kinds of optimization
problems. Specifically Octave can solve problems in Linear Programming,
Quadratic Programming, Nonlinear Programming, and Linear Least Squares
Minimization.

- Linear Programming- Quadratic Programming- Nonlinear Programming- Linear Least Squares
